# Multiprofessional teams (eMulti): potentialities and challenges for the expansion of primary health care in Brazil

**DOI:** 10.1590/0102-311XPT120123

**Published:** 2023-11-13

**Authors:** José Patrício Bispo, Erika Rodrigues de Almeida

**Affiliations:** 1 Instituto Multidisciplinar de Saúde, Universidade Federal da Bahia, Vitória da Conquista, Brasil.; 2 Ministério da Saúde, Brasília, Brasil.

## Introduction

The recently enacted *Ordinance n. 635*, of May 22, 2023 of the Brazilian Ministry of Health, instituted the federal financial incentive for implementation and funding of multiprofessional teams (eMulti) in primary health care (PHC) ^1^. The new proposal presents interprofessionalism as one of its guidelines and constitutes a substitute arrangement for the Family Health Support Center (NASF).

The eMulti emerge amidst a scenario of reconstruction of PHC in Brazil, with the strengthening of interprofessional actions and in the interface with the agenda of technology and innovation incorporations in health. The new arrangement maintains some similarities with the work of NASF and has new organizational and structural mechanisms. In this context, some issues and characteristics are unclear, raising reflections on this new multi-professionalism model.

This article aims to reflect on the potentialities and challenges of eMulti for the expansion of resolvability in PHC in Brazil. To this end, we took as reference the scenario of reconstruction of the Brazilian Unified National Health System (SUS) ^2^, the context of PHC in Brazil ^3^, and the challenges of multi-professionalism in PHC ^4^.

## Context and characteristics of the eMulti

From 2016 to 2022, two radical right-wing governments, guided by a political vision of rigid fiscal austerity and by the defense of privatizing public services, were installed in Brazil. These governments operated to dismantle the SUS, de-funding healthcare and, consequently, weakening PHC ^2^. This scenario began to change in January 2023, with the inauguration of President Lula and the resumption of the strengthening and consolidation of the SUS.

In the first 100 days of government, the Brazilian Ministry of Health had already adopted relevant measures, such as the repeal of technical notes and ordinances that contradicted science, human rights, and sexual and reproductive rights. The repeal of *Ordinance n. 2,561/2020*, which required health professionals to notify the police about the performance of legal abortion in case of rape, is a relevant example. Other examples of the government’s direction for rebuilding the SUS and for guaranteeing health as a right of citizenship include the resumption of the More Doctors Program; the strengthening of the immunization agenda through the National Movement for Vaccination; the expansion of the coverage of PHC services through the publication of new team accreditations; the resumption of the food and nutrition security agenda; the expansion of Health Residency grants; the sanction of the Brazilian National Oral Health Policy in the Organic Health Law; the regulation of the national nursing minimum salary; and the institution of the financing of the eMulti.

The eMulti are defined as teams of health professionals from different areas who work for PHC in a complementary and integrated manner, acting co-responsibly to benefit the population and territory, in intersectoral coordination, along with the Health Care Network ^1^. There are three different types of eMulti, which can be composed of a fixed or variable set of professionals. The Extended eMulti is linked to 10 to 12 PHC teams and composed of a minimum workload of 300 hours. The Complementary eMulti must support from 5 to 9 teams and be composed of a minimum of 200 working hours. The Strategic eMulti, with a minimum workload of 100 hours, must assist 1 to 4 PHC teams ^1^. Thus, it is relevant to note that there is an opportunity to universalize eMulti for all municipalities in Brazil and even to install an intermunicipal model.

Another characteristic that favors the scope of the eMulti is their possibility to be linked to all modalities of PHC teams. Each multidisciplinary team can be integrated into one or more of the following: family health teams; riverine communities family health teams; street clinic teams; primary care teams; or teams from basic fluvial health units. In turn, PHC teams can only be linked to one eMulti.

The wide range of activities that can be developed by the eMulti is also noteworthy: individual, group and home care; collective activities; matrix support activities; case discussions; shared care between professionals and teams; remote medical consultations; therapeutic projects and interventions in the territory; and intersectoral actions. Depending on how these activities are organized and offered, they can contribute to the expansion of the PHC resolvability, as provided for in the regulations.

## eMulti: possibilities and challenges for PHC advancement

Meeting the demands of people, of population and of territories is the main purpose of the eMulti ^1^. Objectives such as broadening the scope of practices, improving the resolvability of PHC and integrating care, prevention, surveillance and training into health denote the target of the eMulti: comprehensive health care.

Thus, we analyzed certain strategic dimensions of new teams that may constitute potentialities or challenges for the expansion of PHC.

Financial incentive stands out as a potential and a relevant inductive strategy to strengthen interprofessional work in PHC. It is worth noting that what followed the revision of the 2017 Brazilian National Primary Health Care Policy (PNAB) and the publication of the *Constitutional Amendment n. 95/2016* was the limitation of public investments in health ^3^. In 2019, this situation was aggravated by the *Previne Brasil*, which extinguished discretionary funding for NASF teams. As a consequence, the number of NASFs significantly decreased in 2020 and 2021 ^4^.

Given this context, we consider that *Ordinance n. 635/2023* has great capacity to encourage greater municipality adherence, since it presents attractive financial values. An Extended eMulti that obtains high performance and carries out remote consultations can reach, from January 2024, BRL 47,500 per month. When considering only personnel remuneration, to fulfill the 300-hour minimum workload, the equivalent value of a 40-hour working week would be BRL 6,000 and that of a 20-hour working week would be BRL 3,000. Although it is known that the eMulti involves many other costs, these equivalent values are certainly higher than those paid to NASF professionals (except for physicians).

Another positive aspect is the wide diversity of professionals that can compose the teams. According to the ordinance, the eMulti can include professionals from 12 different areas and 11 medical specialties, strengthening interprofessionalism in PHC.

The wide range of activities that can be developed by the eMulti is also noteworthy. The planned actions contribute to the expansion of the scope of practices and resolvability in PHC. Notably, the eMulti extend the attributions provided for the NASF. In the 2017 PNAB, the matrix support activities was removed from the set of actions of NASF. With the eMulti, it was once again included as a formally activity. The incorporation of health actions mediated by information and communication technologies, especially teleconsultations, also has great potential to reduce referrals to other health care levels.

In this sense, we consider that teleconsultations show the innovative character of the proposal and enable the expansion of the scope of health care practices, especially in locations distant from large urban centers and where it is difficult to find specialists. In turn, depending on how this modality is implemented, a fully virtual eMulti may be established, which can damage the bond among teams and between the eMulti and the users. Remote care predominantly covers health care specialties for which there is a lack of medical professionals in certain regions of Brazil. This increases the risk of the emergence of specialized clinics decontextualized from the attribute of community orientation.

A potentiality of the eMulti is the establishment of incentive remuneration, paid based on performance assessment. Instituting performance assessment with indicators aimed at satisfying patients and solving problems strengthens the proposal and signals the possibility of continuous improvement. Moreover, such indicators can support the shaping of the teams’ work agendas and induce a health care model favorable to the consolidation and strengthening of PHC.

A challenge to this system is the current need for better guidance on the organization of the eMulti’s work. The proposal does not clarify what the eMulti are expected to focus on, how the work process should be structured and how agreements with PHC teams should occur. We consider that the new multiprofessional teams lack an identity in terms of their institutional space and attributions within PHC, situation similar to that of the NASF.

Ambiguities related to the concept and practices of multiprofessional teams have persisted since the creation of NASF ^5^. Bispo Júnior & Moreira ^6^ highlight the dichotomy between individual/curative and group/promotional activities in the work of the NASF. Almeida & Medina ^5^ emphasize that the official NASF norms lack clarity regarding expected results: they list a set of functions without tying these to specific activities. Such operational uncertainties are repeated in the eMulti ordinance, which may compromise the goal of increasing resolvability.

Along with the need to establish the eMulti’s main focus, it is essential to establish the parameters for care flows, that is, the itineraries of patients inside the health care system. The normative also does not clarify the mechanisms of interaction with PHC teams. A persistent question is whether the eMulti’s work will be based on the logic of collaborative care, with the predominance of interaction among teams, or if it will occur mainly through referrals to clinical care.

Another challenge concerns structural conditions. In the ordinance, there are no proposals to restructure the physical spaces of basic health units. Remote care should be provided with assistance, in a room intended for the activity and with the intermediation of a health professional ^1^. It is crucial, therefore, to delineate the necessary adaptations and/or structures for this type of service. The other services also require the physical presence of workers in basic health units. Several studies ^6^ ^,^ ^7^ ^,^ ^8^ indicate that structural guarantees influence the practices and results of NASF work. The value of community spaces has already been made clear, but it is still essential to ensure adequate spaces and conditions for the eMulti’s work within basic health units.

We also highlight the challenges inherent in managing the work and education of the eMulti. It is important to be aware of how labor relations will take place in these teams, especially employment relationships and contracts. It would be incoherent to institute a new health care service without addressing and eliminating risks of precarious work conditions, of outsourcing processes and of harmful forms of contract, such as those instituted in social health organizations. The challenges inherent to the training of professionals and the need to institute a permanent education policy for eMulti and PHC teams are also worth mentioning. The ordinance does not address any aspect of training policies, which means that many professionals will start working at the eMulti without any specific training for the job.

## Final considerations

We believe that the resumption of multidisciplinary teams is strategic to promote the integral care of the population and contributes to broadening the scope of practices and resolvability in PHC. However, simply resuming financing is not enough. It is necessary to establish interprofessionalism as a guideline of the SUS and PHC, and to recognize it as such in the Organic Health Law and in the entire legal framework, including in the reformulation of the PNAB.

It is worth noting that the challenges discussed in this article could not all be addressed by *Ordinance n. 635/2023*, which has the specific purpose of instituting funding for the eMulti. In turn, if such challenges are not properly faced, they can hinder the teams’ performance or even make it unfeasible. It is necessary to improve the organization and training processes in the eMulti, as well as the working conditions in PHC. Adequate sizes for eMulti teams must also be established.
